# Atypical Spindle Cell/Pleomorphic Lipomatous Tumor: A Review and Update

**DOI:** 10.3390/cancers16183146

**Published:** 2024-09-13

**Authors:** Jun Nishio, Shizuhide Nakayama, Yoshiro Chijiiwa, Mikiro Koga, Mikiko Aoki

**Affiliations:** 1 Section of Orthopaedic Surgery, Department of Medicine, Fukuoka Dental College, 2-15-1 Tamura, Sawara-ku, Fukuoka 814-0193, Japan; chijiiwa@fdcnet.ac.jp; 2 Department of Orthopaedic Surgery, Faculty of Medicine, Fukuoka University, 7-45-1 Nanakuma, Jonan-ku, Fukuoka 814-0180, Japan; n.shizuhide@gmail.com (S.N.); miki.zxcv@icloud.com (M.K.); 3 Department of Pathology, Faculty of Medicine, Fukuoka University, 7-45-1 Nanakuma, Jonan-ku, Fukuoka 814-0180, Japan; mikikoss@fukuoka-u.ac.jp

**Keywords:** atypical spindle cell/pleomorphic lipomatous tumor, spindle cell lipoma, pleomorphic lipoma, atypical lipomatous tumor, dedifferentiated liposarcoma, pleomorphic liposarcoma, *RB1* deletion

## Abstract

**Simple Summary:**

Atypical spindle cell/pleomorphic lipomatous tumor (ASCPLT) is a novel entity of benign adipocytic neoplasm that can show variable CD34 immunoreactivity, loss of Rb expression, and *RB1* gene deletion. Due to the rarity of the disease, ASCPLT remains poorly understood. This review aims to provide a comprehensive reference, offer valuable insights into the clinicopathological characteristics and pathogenesis, and discuss the differential diagnosis of ASCPLT.

**Abstract:**

Atypical spindle cell/pleomorphic lipomatous tumor (ASCPLT) is a rare and recently described adipocytic neoplasm that primarily occurs in the subcutis of the limbs and limb girdles, particularly of middle-aged adults. It has locally recurrent potential if incompletely excised but no risk for distant metastasis. ASCPLT is histologically similar to spindle cell/pleomorphic lipoma and atypical lipomatous tumor and shows a mixture of atypical spindle cells, adipocytes, lipoblasts, floret-like multinucleated giant cells, and/or pleomorphic cells. It has been recently recognized that ASCPLT can undergo sarcomatous transformation. However, the biological significance of morphological sarcomatous transformation in ASCPLT remains uncertain. Immunohistochemically, the tumor cells show variable expression of CD34, S-100 protein, and desmin. Loss of nuclear Rb expression is observed in the majority of cases. ASCPLT lacks *MDM2* gene amplification but can show *RB1* gene deletion in a significant subset of cases. Complete surgical excision is the treatment of choice. This review provides an overview of the current knowledge on the clinicoradiological features, pathogenesis, histopathology, and treatment of ASCPLT. In addition, we will discuss the differential diagnosis of this new entity.

## 1. Introduction

Atypical spindle cell/pleomorphic lipomatous tumor (ASCPLT) is a rare, benign adipocytic neoplasm composed of atypical spindle cells, adipocytes, lipoblasts, and pleomorphic (multinucleated) cells [1]. In 1994, Dei Tos et al. described six cases of adipocytic neoplasm containing cellular, spindle areas with variable atypia and proposed the term “spindle cell liposarcoma (SCLPS)” as a separate entity [2]. SCLPS was then regarded as an unusual morphological variant of atypical lipomatous tumor (ALT)/well-differentiated liposarcoma (WDLPS). In 2010, Mentzel et al. postulated that SCLPS could possibly represent the atypical counterpart of spindle cell lipoma (SCL) on the basis of clinicopathological and molecular features, including RB transcriptional corepressor 1 (*RB1*) gene deletion and lack of MDM2 proto-oncogene (*MDM2*) amplification [3]. In 2013, Deyrup et al. described SCLPS as a “fibrosarcoma-like lipomatous neoplasm”, with a better prognosis [4]. In 2014, the term “atypical spindle cell lipoma (ASCL)” was proposed by Creytens et al. [5]. The authors indicated that chromosome 13q14 deletions in ASCL were more extensive and complex than in conventional SCL. In 2017, Marino-Enriquez et al. described a large series of 232 well-differentiated adipocytic neoplasms histologically resembling SCL but showing mild-to-moderate nuclear atypia and increased cellularity and proposed the term “atypical spindle cell lipomatous tumor (ASCLT)”, with generally benign behavior [6]. Meanwhile, Creytens et al. described a series of 21 low-grade adipocytic neoplasms histologically resembling pleomorphic lipoma (PL) but showing nuclear pleomorphism and hypercellularity and proposed the term “atypical pleomorphic lipomatous tumor (APLT)” [7]. The authors demonstrated a significant overlapping of morphological and genetic features between ASCLT and APLT. Currently, ASCLT and APLT are recognized as representing a single entity, namely ASCPLT, in the latest World Health Organization (WHO) classification of soft tissue and bone tumors [1]. The etiology of this neoplasm is unknown. Advances in knowledge of the imaging, histopathology, and genomics of ASCPLT are leading to more accurate diagnosis and appropriate treatment. In this article, we review the clinicoradiological, histopathological, and molecular features of ASCPLT and summarize the current treatment. In addition, we will discuss the differential diagnosis of this recently described entity.

## 2. Clinical Features

ASCPLT primarily occurs in middle-aged adults, with a peak incidence in the sixth decade of life and shows a male predominance [6,7,8]. It typically presents as a persistent or enlarging, painless, superficial soft tissue mass in the limbs and limb girdles. Deep (subfascial) soft tissue involvement can be seen. ASCPLT may also occur in less common locations such as the head and neck, trunk, and groin [6,7,8,9]. The median tumor size is 5 cm, with a range from 0.5 to 28 cm [6]. The clinical presentation of ASCPLT is variable and depends on the location of the tumor. The median preoperative duration of symptoms is 4 months [8].

Importantly, ASCPLT pursues a benign clinical course with a low recurrence rate of 4–17% for incompletely excised lesions [6,7,8]. Distant metastasis has not been documented. Recently, however, five cases of ASCPLT with unequivocal sarcomatous transformation have been described in the literature [10,11] (Table 1). In 2022, Sugita et al. reported a single case of ASCPLT with a sarcomatous component showing high mitotic activity and Ki-67 labeling index (LI), mimicking dedifferentiated liposarcoma (DDLPS) [10]. The patient was alive with no evidence of local recurrence or distant metastasis at 3.5 years after wide excision. In 2024, Perret et al. reported four cases of ASCPLT with a high-grade sarcomatous component displaying more complex genomic profiles [11]. Limited clinical follow-up showed no sign of local recurrence or distant metastasis at 1–13 months after surgical excision with positive margins (conventional ASCPLT component only). Further studies are needed to better understand the clinical behavior of these rare cases.

## 3. Imaging Features

There is only a very limited description of the imaging appearance of ASCPLT. In our opinion, distinguishing ASCPLT from other lipomatous tumors such as SCL/PL and ALT can be challenging based on imaging features alone and often requires histological and molecular evaluations.

Radiographs may be normal or reveal a non-specific soft tissue mass. Computed tomography (CT) may show a solid, heterogeneous contrast-enhanced mass [12,13,14]. On magnetic resonance imaging (MRI), ASCPLT shows variable signal intensity on T1- and T2-weighted images [10,13,14,15,16,17]. Contrast-enhanced MRI demonstrates heterogeneous enhancement of the lesion. Interestingly, Sugita et al. reported a unique case of ASCPLT consisting of an adipocytic component and a non-adipocytic component on MRI, suggesting a diagnosis of DDLPS [10]. To date, position emission tomography (PET) features for ASCPLT have been described in only one case [16]. Integrated PET/CT images show slight fluorodeoxyglucose (FDG) uptake in the lesion, with the maximum standardized uptake value (SUVmax) of 2.8.

## 4. Pathogenesis

Cytogenetically, a partial or complete monosomy 7 was detected in two cases [18]. Subsequently, fluorescence in situ hybridization (FISH) analysis with the chromosome 7 centromeric probe confirmed the presence of monosomy 7 in three (43%) of the seven cases [6]. In addition to loss of chromosome 7, array comparative genomic hybridization (CGH) analysis has shown recurrent losses of chromosomes 4, 6q, 8p, 10, 13q, 16q, 17p, 17q, and 20p [7,19]. The most common recurrent loss is found in chromosome arm 13q.

The deletion of *RB1*, located at chromosome 13q14.2, has been identified in a significant subset of cases [5,6,7]. *RB1* is an important tumor suppressor gene involved in the cell cycle. Loss of Rb expression and the *RB1* gene can be detected using immunohistochemistry and FISH, respectively [20]. It is of interest that *RB1* deletion has also been reported as a recurrent finding in other soft tissue tumors, including SCL/PL, pleomorphic liposarcoma (PLPS), myxoid pleomorphic liposarcoma (MPLPS), myofibroblastoma, cellular angiofibroma, and acral fibromyxoma [21,22]. Additionally, the deletion of forkhead box O1 (*FOXO1*), located at chromosome 13q14.11, was detected in 19 (90.5%) of 21 cases [23]. FOXO1 is also considered a tumor suppressor and plays crucial roles in cell cycle control, apoptosis, metabolism, and adipocyte differentiation [24]. Zhang et al. reported that the deletion rate of co-hybridized *RB1* and *FOXO1* was 85.7% [23]. Moreover, it has been recognized that chromosome 13q deletion in ASCPLT is more extensive and complex than in SCL/PL, including *RB1* and its flanking genes (integral membrane protein 2B (*ITM2B*), RCC1 and BTB domain-containing protein 2 (*RCBTB2*), and deleted in lymphocytic leukemia 1 (*DLEU1*)) [5,7]. Recently, however, Creytens et al. reported that the loss of *RB1* and its flanking genes (*ITM2B*, *RCBTB2*, and *DLEU1*) was seen in four (50%) of the eight analyzed MPLPSs [22].

It is noteworthy that ASCPLT displays a consistent absence of *MDM2* amplification [6,7,8,9]. Importantly, the diagnosis of ASCPLT should be avoided if *MDM2* gene amplification is shown.

In 2022, Hammer et al. investigated tumor protein p53 (*TP53*) abnormalities in six ASCPLT cases using massively parallel sequencing [25]. *TP53* mutation or deletion was found in four (67%) of the six cases. Most recently, Perret et al. reported that the deletion of *TP53* was seen in both conventional and sarcomatous components of ASCPLT [11]. *TP53* encodes a tumor suppressor protein containing transcriptional activation, DNA binding, and oligomerization domains. These findings suggest that *TP53* alterations may contribute to the pathogenesis of ASCPLT in at least a subset of cases.

## 5. Histopathology

Grossly, ASCPLT usually appears as an ill-defined, unencapsulated, nodular/multinodular mass with a tan-yellow lobulated cut surface [1,7,8,9]. Small foci of hemorrhage may be seen but necrosis is absent [8].

Histologically, ASCPLT typically shows infiltrative margins and can demonstrate a wide range of appearances, even regionally within the same lesion [1]. ASCPLT is composed of variable proportions of atypical spindle cells, predominantly mature adipocytes, lipoblasts, and floret-like multinucleated giant cells in a collagenous and/or myxoid matrix. In addition, bizarre, pleomorphic (often multinucleated) cells may be present [7]. Unlike SCL/PL, the presence of refractile ropey collagen bundles is rare [6]. Mitotic activity is very low, ranging from 1 to 2 mitoses per 50 high-power fields [6,9]. Atypical mitoses are unusual and tumor necrosis is absent. Heterogeneous differentiation is rare and includes smooth muscle, osseous, and cartilaginous elements [6,26]. Interestingly, fat-rich and pleomorphic hyalinizing angiectatic tumor-like growth patterns have been described in ASCPLT [27,28].

Hitherto, there are several reported cases of ASCPLT resembling a phenomenon of dedifferentiation [6,9] or displaying a sarcomatous transformation [10,11]. In two previous series of ASCPLT, three cases showed an abrupt transition from lipogenic to non-lipogenic spindle cell areas with increased cellularity and nuclear atypia [6,9]. Follow-up available in two cases revealed no sign of local recurrence or distant metastasis at 97 and 37 months. In a case report and small case series of ASCPLT, five cases showed a biphasic appearance comprising a conventional ASCPLT component and a high-grade sarcomatous component with brisk mitotic activity and necrosis [10,11]. The high-grade components showed leiomyosarcoma-like (*n* = 2), undifferentiated sarcoma-like (*n* = 2), and PLPS-like (*n* = 1) morphology. In addition, Ki-67 LI in the sarcomatous component reached 40% in one case [10]. Intriguingly, the sarcomatous components displayed more complex genomic profiles with numerous chromosomal aberrations, compared with the conventional components [11].

Immunohistochemically, the tumor cells show variable expression of CD34, S-100 protein, and desmin [6,8]. Although non-specific, nuclear p16 expression is also observed, especially in APLT [7]. Notably, a loss of nuclear Rb expression is seen in 57–79% of cases [6,8], in accord with the deletion of chromosome 13q14 including the *RB1* gene. Weak and/or focal expression of MDM2 or cyclin-dependent kinase 4 (CDK4) may be seen but co-expression of MDM2 and CDK4 is absent [1,6].

## 6. Management

In general, a preoperative biopsy is recommended for large (>10 cm diameter) or deep-situated adipocytic tumors, including ASCPLT. In our opinion, a pretreatment histopathological diagnosis should be made by biopsy when imaging is suspicious. A planned excision biopsy may be the most practical option for small (<2 cm diameter) subcutaneous lesions that are indeterminate on imaging.

Surgical excision is the mainstay of treatment for ASCPLT. The surgical procedure is complete excision with negative margins. Local recurrence is not aggressive and typically responds to re-excision.

There is a question as to whether ASCPLT with sarcomatous component needs to be widely excised. As mentioned above, Sugita et al. reported that one patient with sarcomatous component underwent wide excision because of a high suspicion of DDLPS and was alive with no evidence of local recurrence or distant metastasis 3.5 years after surgery [10]. The authors suggested that wide excision might be considered if a preoperative diagnosis of ASCPLT with sarcomatous component is made. Perret et al. also reported that all four patients with sarcomatous transformation underwent surgical excision, and the margins were positive by the adipocytic component only in all cases [11]. Re-excision was only performed in one case and all patients were alive with no evidence of disease at the last clinical follow-up. Additional studies are required to better define an adequate margin of excision for ASCPLT with sarcomatous component.

Radiation therapy (RT) can be used as a perioperative treatment strategy to improve local disease control. There are few reports regarding the use of perioperative RT in patients with ASCPLT [6,8]. In our opinion, most patients with ASCPLT will not require RT. However, RT may be considered for patients with positive surgical margins if re-excision is not possible. Currently, the role of RT in the treatment of ASCPLT is controversial.

## 7. Differential Diagnosis

The differential diagnosis for ASCPLT is broad due to a wide variety of histological appearances and should include benign, intermediate, and malignant adipocytic neoplasms [29,30]. In our extensive experience, ASCPLT is most often confused with SCL/PL and ALT/WDLPS. Moreover, PLPS/MPLPS can be challenging to differentiate from ASCPLT, especially in small tissue samples. The corresponding clinicopathological and molecular characteristics are summarized in Table 2.

SCL and PL represent the morphological spectrum of a single entity [31]. SCL/PL is a benign adipocytic neoplasm that primarily occurs in middle-aged adults, with a peak incidence in the fifth to seventh decades of life, and shows a marked male predominance. Unlike ASCPLT, the vast majority of SCL/PLs occur in the posterior neck, shoulder, and upper back. SCL/PL typically presents as a usually solitary, mobile, slowly growing, painless, subcutaneous mass. The diameter ranges from 1 to 14 cm (usually less than 5 cm) [32]. The imaging appearance of SCL/PL is not pathognomonic [33] and may overlap with that of ASCLPT. Integrated PET/CT images show increased FDG uptake in the non-adipocytic component, with an SUV range of 2–8 [34]. Surgery is the treatment of choice for SCL/PL. Local recurrence is very rare and there is no metastatic potential. Histologically, SCL/PL shares many of the morphological features of ASCPLT and is composed of bland spindle cells and mature adipocytes in a fibromyxoid stroma. Floret-like multinucleated giant cells and pleomorphic spindle cells can be seen in PL [31], resembling ASCPLT. Unlike ASCPLT, the presence of ropey collagen bundles is very common, and spindle cells with nuclear atypia/hyperchromasia and bizarre-looking lipoblasts are absent. Mitotic figures are rare and tumor necrosis is absent. SCL/PL and ASCPLT share similar immunohistochemical features, including CD34 positivity and loss of nuclear Rb expression [32]. Positive staining for desmin and S-100 protein may be seen in a subset of cases [35,36]. Cytogenetically, SCL/PL is characterized by deletions of partial or whole chromosome 13 and/or 16 [31,37,38]. Like ASCPLT, the heterozygous deletion of *RB1* and/or *FOXO1* has been detected in a significant subset of cases [37]. These findings indicate that the discrimination between ASCPLT and SCL/PL cannot be based on immunohistochemical and molecular features.

According to the latest WHO classification of soft tissue and bone tumors, ALT is synonymous with WDLPS [39]. The use of the term ALT is determined principally by tumor location and resectability. ALT is an intermediate adipocytic neoplasm that primarily occurs in middle-aged adults, with a peak incidence in the fourth to fifth decades of life and shows no gender predominance [39]. It usually presents as a slowly growing, painless, deep-seated soft tissue mass in the lower extremities. The retroperitoneum and trunk are also commonly involved. Unlike ASCPLT, superficial soft tissue involvement is rarely seen. The diameter ranges from 2 to 35 cm (median of 18 cm) [40]. On CT and MRI, ALT reveals a predominantly adipocytic mass with thick septa and/or a nodular or patchy non-adipocytic component. Contrast-enhanced CT and MRI demonstrate significant enhancement of the septa and non-adipocytic component [40]. Integrated PET/CT images show low FDG uptake in the lesion, with the SUVmax range of 0.8–3.9 [41]. In our opinion, differentiating between ASCPLT and ALT may be difficult even after good MRI due to their similar imaging appearance. The standard treatment for ALT is surgery. As previously suggested, we believe that marginal excision is a reasonable surgical approach for deep-seated ALT of the extremities [40]. The local recurrence rate following marginal excision is 8.4–23% [42,43,44], but local recurrence can be managed with re-excision. Deep-seated ALT of the extremities shows no potential for distant metastasis unless it undergoes dedifferentiation. The ultimate risk of dedifferentiation is probably less than 2% in the extremities [39]. Histologically, ALT shows the greatest morphological overlap with ASCPLT [30] and is composed of variably sized mature adipocytes and atypical, hyperchromatic stromal spindle cells. A variable number of lipoblasts can be seen. Compared with ASCPLT, however, pleomorphic lipoblasts are rather rare in ALT [29]. In the inflammatory subtype, an abundant chronic inflammatory infiltrate is found. Mitotic figures are uncommon. Immunohistochemically, in contrast to ASCPLT, expression of MDM2 and/or CDK4 is seen in the vast majority of ALTs [39]. Cytogenetically, ALT is characterized by the presence of supernumerary ring chromosomes and/or giant marker chromosomes. Both ring and giant marker chromosomes are mainly composed of amplified sequences of 12q14-15 including the *MDM2* gene [40]. Most importantly, detection of *MDM2* amplification by FISH can serve to distinguish ALT from ASCPLT [6,7,8].

DDLPS is a malignant adipocytic neoplasm that primarily occurs in middle-aged and older adults, with a peak incidence in the sixth to seventh decades of life and shows no gender predominance [45,46]. It has a broad anatomical distribution, with the retroperitoneum as the most common location. Other locations include the extremities, spermatic cord, trunk, and head and neck [45]. Unlike ASCPLT, superficial soft tissue involvement is rarely seen. DDLPS usually presents as a large, painless, soft tissue mass, which might be found incidentally in the retroperitoneum. The lesion is typically larger than ASCPLT lesion. Importantly, DDLPS is characterized as a typically non-adipocytic sarcoma that is juxtaposed with ALT. On CT, DDLPS usually displays a well-defined mass with slightly increased attenuation compared with that of subcutaneous fat in the adipocytic component and tissue attenuation similar to or slightly lower than that of skeletal muscle in the non-adipocytic component [47]. Contrast-enhanced CT demonstrates significant enhancement in the non-adipocytic component. MRI typically reveals the co-existence of adipocytic (ALT) and juxtaposed non-adipocytic (dedifferentiated) components [46]. The non-adipocytic component usually displays low-to-intermediate signal intensity on T1-weighted images and intermediate-to-high signal intensity on T2-weighted images. Contrast-enhanced MRI demonstrates variable enhancement in the non-adipocytic component. Integrated PET/CT images show a biphasic pattern with a close relationship to MRI features, with the SUVmax range of 1.9–22.6 in the non-adipocytic component [48]. Wide excision is the standard treatment for DDLPS. Local recurrences occur in at least 40% of cases [45]. Unlike ASCPLT and ALT, DDLPS has a metastatic potential, with 15–30% of cases [46]. Overall mortality at 5 years is estimated to be about 28–30% [45]. Histologically, DDLPS usually shows an abrupt transition between ALT and non-adipocytic components. The non-adipocytic component exhibits a variety of morphological appearances. Compared with ASCPLT, pleomorphic lipoblasts are rare but tumor necrosis can be seen in DDLPS. Immunohistochemically, diffuse nuclear expression of MDM2, CDK4, and p16 is seen in the vast majority of DDLPSs [49]. Cytogenetically, like ALT, DDLPS is characterized by the presence of supernumerary ring chromosomes and/or giant marker chromosomes. These ring and giant marker chromosomes are mainly composed of amplified sequences of 12q14-15, with copy number gains of *MDM2* and *CDK4* genes [46]. In addition to the 12q14-15 amplification, high-level amplifications of 1p32 and 6q23 are seen in DDLPS [50]. Importantly, the detection of *MDM2* amplification by FISH is helpful to differentiate DDLPS from ASCPLT.

PLPS is an extremely rare, highly malignant adipocytic neoplasm that primarily occurs in older adults, with a peak incidence in the seventh decade of life, and shows a slight male predominance [51]. The anatomical distribution is similar to ASCPLT, with the extremities being the most common location. Other locations include the trunk, retroperitoneum, head and neck, and spermatic cord [52]. PLPS usually presents as a rapidly growing, usually large, painless, deep-seated soft tissue mass. Approximately 25% of cases arise in the subcutis [53]. Presenting symptoms are typically related to the location of tumor. The median size of PLPS is 8–10 cm [51] and is typically larger than that of ASCPLT. The imaging appearance of PLPS is not pathognomonic and may overlap with that of other high-grade sarcomas [54]. On CT and MRI, PLPS usually shows a relatively well-circumscribed mass, although infiltrative margins may be seen [55,56]. Approximately 75% of cases demonstrate foci of fat on MRI [55]. The signal intensity of the adipocytic component may be slightly less than that of subcutaneous fat on T1-weighted images [55]. The non-adipocytic component usually displays intermediate signal intensity on T1-weighted images and intermediate-to-high signal intensity on T2-weighted images. Integrated PET/CT images show high FDG uptake in the lesion, with the SUVmax range of 11.4–15.5 [41]. Wide excision, with or without RT, is the mainstay of treatment for PLPS. Local recurrences occur in 25–45% of cases [52,53,57]. Unlike ASCPLT, PLPS has a significant metastatic potential in 32–42.5% of cases [52,53,57]. The overall 5-year survival rate is approximately 60% [57]. Histologically, PLPS is poorly circumscribed with infiltrative margins and contains a varying proportion of pleomorphic lipoblasts. Epithelioid morphology is seen in about 25% of cases [51]. Compared to ASCPLT, PLPS tends to have high cellularity, marked pleomorphism, and high mitotic activity. Tumor necrosis is found in more than half of cases [51]. Immunohistochemically, PLPS is focally positive for smooth muscle actin (SMA), CD34, S-100 protein, and desmin [52,53]. The epithelioid subtype may be positive for keratin [58]. It should be noted that PLPS may show a loss of nuclear Rb expression, similar to ASCPLT [7]. Staining for MDM2 and CDK4 is typically negative. Cytogenetically, PLPS is characterized by complex karyotypes without pathognomonic structural alterations [59]. Array CGH analysis has shown losses of 1q, 2q, 3p, 4q, 10q, 11q, and 13q and gains of 5p, 19p, 19q, and 20q [60]. Like ASCPLT, the most common recurrent loss is seen in chromosome band 13q14-21. Interestingly, loss of *RB1* has been identified in a subset of cases [7], suggesting a genetic overlap between PLPS and ASCPLT. Moreover, *TP53* and neurofibromin 1 (*NF1*) mutations have been detected in 17% and 8% of cases, respectively [61]. The amplification of 12q14-15 containing *MDM2* and *CDK4* is absent in PLPS [62].

MPLPS is an exceedingly rare, highly aggressive adipocytic neoplasm that primarily occurs in children, adolescents, and young adults and shows a female predominance [63]. Unlike ASCPLT, MPLPS has a strong predilection for the mediastinum. Other less common locations include the neck, orbit, cheek, pleura, perineum, abdomen, back, and thigh [64]. MPLPS usually presents as a large, deep-seated soft tissue mass with ill-defined margins. The median tumor size is 12.5 cm, with a range from 4.8 to 18 cm [65]. Notably, there is an association between MPLPS and Li–Fraumeni syndrome [66,67]. Current understanding of the imaging appearance of MPLPS is limited. On CT, MPLPS may show a hypodense mass with heterogeneous enhancement [65,68,69]. MRI may reveal a heterogeneous soft tissue mass with adipocytic and myxoid components [65,70]. Surgical excision is the mainstay of treatment for MPLPS. The role of RT and chemotherapy remains controversial. Local recurrences occur in 40–50% of cases [22,64,65]. Unlike ASCPLT, MPLPS has a high metastatic potential in 40–100% of cases [22,64,65]. Overall mortality at 52 months is estimated to be about 74% [65]. Histologically, the hallmark of MPLPS is the presence of hybrid morphological features resembling conventional myxoid liposarcoma (MLPS) and PLPS [68]. The MLPS-like areas are composed of relatively uniform and bland ovoid-to-spindle cells in an abundant myxoid stroma with a well-developed capillary vasculature. The PLPS-like areas display marked nuclear atypia, increased mitotic activity with atypical mitoses, and pleomorphic lipoblasts. Occasionally, tumor necrosis is found in the background. In general, immunohistochemistry plays little role in the diagnosis of MPLPS [63]. Similar to ASCPLT, diffuse expression of CD34 and p16 and loss of nuclear Rb expression have been observed in all cases examined [22]. Staining for MDM2 and CDK4 is typically negative [22,64]. Cytogenetic and array CGH analyses and genome-wide copy number profiling have shown complex chromosomal alterations including gains in chromosomes 1, 6–8, 18–21, and X and losses in chromosomes 2–5, 10–17, and 22 [22,71,72]. In addition, allele-specific copy number profiling has demonstrated a widespread loss of heterozygosity (approximately 80% of the genome on average) [65]. Like ASCPLT, the loss of 13q14 and monoallelic *RB1* deletion have been observed in 50% and 67% of cases, respectively [22]. Moreover, *TP53* mutations have been detected in all cases examined [65]. MPLPS lacks both DNA damage-inducible transcript 3 (*DDIT3*) rearrangement characteristic of MLPS and *MDM2* amplification [22,73,74].

## 8. Conclusions and Future Directions

ASCPLT is a benign adipocytic neoplasm with a low but non-negligible risk for local recurrence and no metastatic risk that predilects the superficial soft tissues of the limbs and limb girdles in middle-aged adults. The diagnosis of ASCPLT can be challenging due to its overlapping clinicoradiological, morphological, and molecular features. Histologically, ASCPLT consists of atypical spindle cells, predominantly mature adipocytes, lipoblasts, floret-like multinucleated giant cells, and/or pleomorphic cells. Notably, it should be kept in mind that ASCLPT can undergo sarcomatous transformation. Identification of CD34 immunopositivity, loss of Rb expression or *RB1* gene deletion, and lack of *MDM2* amplification would be helpful diagnostically for ASCPLT in selected cases. Surgery is the mainstay of treatment for localized ASCLPT, and local recurrence can usually be managed with re-excision. Further studies with a large number of cases are needed to better understand the correlation between morphological sarcomatous transformation and distinct biological behavior.

## Figures and Tables

**Table 1:** Clinical features of 5 cases of ASCPLT with a sarcomatous component.

Case	Age/Gender	Site/Size (cm)	Treatment/Margin Status	Outcome	Follow-Up Period (Months)	Reference
1	78/F	Thigh/12.2	Wide excision/Negative	AWED	42	Sugita et al. [10]
2	74/M	Trunk/8	Primary excision and re-excision/Negative	AWED	13	Perret et al. [11]
3	65/F	Arm/10.5	Excision/Positive (conventional adipocytic component only)	AWED	6	Perret et al. [11]
4	78/M	Trunk/5.5	Excision/Positive (conventional adipocytic component only)	AWED	9	Perret et al. [11]
5	70/M	Arm/3	Excision/Positive (conventional adipocytic component only)	AWED	1	Perret et al. [11]

ASCPLT: atypical spindle cell/pleomorphic lipomatous tumor; F: female; M: male; AWED: alive without evidence of disease.

**Table 2:** Differential diagnosis of ASCPLT.

Tumor	Age/Gender	Predilection Site	Prognosis	Histopathology	Molecular Features
ASCPLT	Middle-aged adults; male predominance.	Limb girdles, limbs.	Low LR rates; no metastatic potential.	Ill-defined margins; lipoblasts (+), pleomorphic multinucleated cells (+), no necrosis, CD34 (+), desmin (variable), S-100 protein (variable), Rb loss.	*RB1* deletion; no *MDM2* amplification.
SCL/PL	Middle-aged adults; strong male predominance.	Posterior neck, back, shoulder.	Very low LR rates; no metastatic potential.	Well-defined margins; ropy collagen bundles (+), floret-like multinucleated giant cells (+), no necrosis, CD34 (+), Rb loss.	*RB1* deletion; no *MDM2* amplification.
ALT	Middle-aged adults; no gender predominance.	Extremities.	Relatively low LR rates; no metastatic potential (unless ALT undergoes dedifferentiation).	Well-defined margins; lipoblasts (variable), atypical stromal spindle cells (+), no necrosis, MDM2 (+), CDK4 (+).	*MDM2* amplification; no *RB1* deletion.
DDLPS	Middle-aged and older adults; no gender predominance.	Retroperitoneum, extremities.	High rates of LR and distant metastasis.	Ill-defined margins; lipoblasts (+), pleomorphic multinucleated cells (variable), occasional necrosis, MDM2 (+), CDK4 (+).	*MDM2* amplification; no *RB1* deletion.
PLPS	Older adults; slight male predominance.	Extremities.	High rates of LR and distant metastasis.	Ill-defined margins; pleomorphic lipoblasts (+), pleomorphic multinucleated cells (+), frequent necrosis, Rb loss, keratin (+) in epithelioid subtype.	*RB1* deletion; no *MDM2* amplification.
MPLPS	Children, adolescents, and young adults; female predominance.	Mediastinum.	High rates of LR and distant metastasis.	Ill-defined margins; pleomorphic lipoblasts (+), pleomorphic multinucleated cells (+), chicken wire capillary vasculature (+), occasional necrosis, Rb loss.	*RB1* deletion; no *MDM2* amplification; no *DDIT3* rearrangement.

ASCPLT: atypical spindle cell/pleomorphic lipomatous tumor; SCL/PL: spindle cell lipoma/pleomorphic lipoma; ALT: atypical lipomatous tumor; DDLPS: dedifferentiated liposarcoma; PLPS: pleomorphic liposarcoma; MPLPS: myxoid pleomorphic liposarcoma; LR: local recurrence; MDM2: MDM2 proto-oncogene; CDK4: cyclin-dependent kinase 4; *RB1*: RB transcriptional corepressor 1; *DDIT3*: DNA damage-inducible transcript 3.
